# Exploring the Nexus between Moderate-to-Vigorous Physical Activity, Self-Disclosure, Social Anxiety, and Adolescent Social Avoidance: Insights from a Cross-Sectional Study in Central China

**DOI:** 10.3390/children11010056

**Published:** 2023-12-30

**Authors:** Shuyin Chen, Longjun Jing, Chuchu Li, Huilin Wang

**Affiliations:** 1 School of Physical Education, Hunan University of Science and Technology, Xiangtan 411201, China; 2 China Athletics College, Beijing Sport University, Beijing 100061, China; 3 Moray House School of Education and Sport, The University of Edinburgh, Edinburgh EH8 8AQ, UK; 4 School of Business, Hunan University of Science and Technology, Xiangtan 411201, China

**Keywords:** adolescents, moderate-to-vigorous physical activity, social anxiety, self-disclosure, social avoidance

## Abstract

**Objectives:** Psychological issues among adolescents represent a prevalent challenge in today’s society. The purpose of this study is to explore the associations among moderate-to-vigorous physical activity, self-disclosure, social anxiety, and social avoidance in adolescents. **Methods:** This study collected cross-sectional data from 427 students in eight provincial key junior and senior high schools in the central China region of three provinces using snowball sampling and convenience sampling from July to August 2023. A structural equation model was employed to investigate the relationship between moderate-to-vigorous physical activity and social avoidance among adolescents. **Results:** The findings indicate that moderate-to-vigorous physical activity is negatively correlated with social anxiety (standardized coefficient = −0.219, *p* < 0.001) and positively correlated with self-disclosure (standardized coefficient = 0.454, *p* < 0.001). Social anxiety is negatively correlated with self-disclosure (standardized coefficient = −0.220, *p* < 0.001). Social avoidance is positively correlated with social anxiety (standardized coefficient = 0.461, *p* < 0.001) and negatively correlated with self-disclosure (standardized coefficient = −0.331, *p* < 0.001). **Conclusions:** The chain-mediated dual-path model between moderate-to-vigorous physical activity and social avoidance is facilitated by social anxiety and self-disclosure. In other words, adolescents who engage in more moderate to high-intensity physical activities exhibit lower levels of social anxiety, and those who have a stronger inclination for self-disclosure tend to demonstrate lower levels of social avoidance. In light of these findings, it is recommended that the government, society, schools, and families collaborate synergistically to promote the holistic well-being of adolescents and advance the development of a healthier China.

## 1. Introduction

Adolescent health and well-being are crucial for the future of a nation and its people, forming the foundation for individual health and happiness. Adolescents commonly face psychological challenges such as social anxiety, social avoidance, depression, anxiety, and low self-esteem [1]. According to the World Health Organization, the global prevalence of mental disorders among children and adolescents ranges from 12% to 28%, showing an upward trend. The Chinese National Report on Mental Health Development (2019–2020) from the Chinese Academy of Sciences Institute of Psychology reveals that psychological health issues among Chinese children and adolescents range from 5% to 30%, with approximately one in five adolescents experiencing some level of mental health problem. In 2020, the detection rate of depression among Chinese adolescents was 24.6%. These data indicate significant issues with adolescent mental health, drawing widespread attention and research. It is imperative for all stakeholders to play their roles in devising scientific and feasible measures for its relief and prevention.

Adolescence, as a transitional phase, exposes individuals to significant social challenges and psychological pressures. Effective coping during this period is a crucial test for adolescents entering a new societal paradigm [2]. Social avoidance in adolescents can be attributed to various factors: (1) Genetic predisposition: research suggests that 60% of social avoidance personality traits are influenced by familial genetic factors. (2) Environmental influences: approximately 30% of adolescent personality development is influenced by the environment, manifested in either inadequate parental care or excessive parental control. Adolescents are often subjected to high-pressure environments, bearing external responsibilities and expectations. (3) Psychological resilience: adolescent psychological resilience is compromised when childhood needs are not met, leading to frequent experiences of denial, ridicule, and humiliation. Research indicates that the emergence of social avoidance from early childhood to adolescence reflects challenges in peer relationships, weak psychological resilience, difficulties in emotional expression, and social adaptation. Consequently, utilizing social avoidance as an outcome variable serves as a means to reflect and address adolescent mental health issues.

Engaging in regular physical activity over an extended period is a powerful method for promoting both physical and mental health [3]. The degree of benefits to physical health varies with different intensities and patterns of exercise [4]. In terms of exercise intensity, moderate-to-vigorous physical activity has significant effects on weight loss [5] and on lowering blood pressure [6], improving physical fitness in adolescents [7], increasing social skills [8], heightening self-efficacy [9], and reducing disease risk [10]. From a physiological perspective, physical exercise can promote the release of dopamine and endorphins, enhance insulin resistance [11], and help reduce the occurrence of negative emotions such as social anxiety and avoidance, thereby encouraging a positive approach to life and reducing tendencies toward social avoidance behavior [12]. In addressing the health and growth of adolescents, the General Administration of Sport and the Ministry of Education in China issued a notice in 2020 titled “Opinions on Deepening the Integration of Physical Education and Teaching to Promote the Healthy Development of Adolescents.” This emphasizes monitoring and intervening in the mental and physical health of adolescents during childhood, fostering robust physical and character development through physical activity. Longitudinal studies confirm that the fear of negative evaluation and the avoidance of adolescents can affect their level of popularity and likability among others [13]. Social avoidance may directly impact a person’s social status more than social anxiety, as people can observe others’ behavior, while thoughts cannot be intuitively perceived.

Epidemiological surveys reveal an 8.89% prevalence of social anxiety among adolescents, higher than the rates observed in domestic and international students during childhood and adolescence. Social anxiety and social avoidance represent different manifestations at the psychological and behavioral levels. Social avoidance intensifies during the 7 to 12 years of adolescence, potentially hindering adolescents’ ability to balance independence and interdependence [14]. Research by Stentz and Cougle [15] suggests that individuals with social anxiety may influence the intimacy of interpersonal relationships by reducing self-disclosure and increasing vigilance, indicating a negative correlation between social anxiety and self-disclosure. However, a study by Chan found a positive correlation between social anxiety, self-disclosure, and happiness on social media platforms, suggesting that increased social anxiety leads to more self-disclosure and, in turn, greater happiness. This insight contributes to providing feasible recommendations for entrepreneurs, educators, doctors, and other professionals.

Given the correlations between social anxiety, self-disclosure, and the impact of moderate-to-vigorous physical activity on adolescent social avoidance, it is reasonable to assume that relationships exist among these variables. The objective of this study is to explore the impact of moderate-to-vigorous physical activity on adolescent social avoidance, with social anxiety and self-disclosure as mediating factors. The ultimate goal of this research is to reduce social problems among adolescents and enhance their satisfaction with life and learning. Specifically, the objectives are the following: (1) to understand social avoidance behaviors in Chinese adolescents; (2) to investigate the relationships among moderate-to-vigorous physical activity, social anxiety, self-disclosure, and social avoidance in adolescents; and (3) to provide feasible recommendations to the government and society based on the identified issues in adolescent social interactions.

The contributions of this study are as follows: from a theoretical perspective, adolescence is a transitional period from childhood to adulthood, representing a population full of dreams and hopes. This research explores the indirect association between moderate-to-vigorous physical activity and social avoidance in adolescents, using path analysis to investigate the mediating roles of social anxiety and self-disclosure. The study discovers that moderate-to-vigorous physical activity influences social avoidance behaviors in adolescents. Beyond the existing analyses, this research further examines the impact of moderate-to-vigorous physical activity on adolescent mental health, providing a theoretical basis for addressing adolescent mental health issues globally. A unique aspect of this study is treating social avoidance as the target variable, as opposed to a mediator or moderator, distinguishing it from previous research approaches.

## 2. Literature Review and Hypotheses Development

### 2.1. Moderate-to-Vigorous Physical Activity, Social Anxiety, and Self-Disclosure

The definition of physical activity can be elucidated from both macro and micro perspectives, encompassing competitive sports activities focused on competition, improving athletic performance, or mastering sports skills [16] and physical activities aimed at enhancing health and well-being [17,18]. Currently, individuals self-assess the intensity of their physical activity using the widely recognized Daily Activity Borg Scale (DABS). This scale is based on the participants’ perceived physical activity over the past 24 h (in hours) and is rated on a 1–9 scale within the exercise range. Comparing the results with the perceived and reported actual physical activity in the last 24 h allows for a more direct, scientific, and effective determination of the intensity of human physical activity [19].

The intensity of physical activity is typically expressed in metabolic equivalents (METs), where 1.5–3 METs represent light physical activity, 3–6 METs represent moderate-intensity physical activity, and greater than 6 METs represent high-intensity physical activity [20]. The benefits of moderate-to-vigorous physical activity include the following: Firstly, in disease treatment, many countries, international health organizations, and scientific associations have confirmed that physical activity can serve as a therapeutic tool for obesity, diabetes, and preventing cardiovascular diseases [21]. The American College of Sports Medicine found it effective in treating symptoms arising from substance use disorders (SUDs) [22]. Secondly, in terms of physical health, the more sports activities school-age children and adolescents engage in, and the higher the intensity, the greater the benefits to their health [23]. In adhering to moderate-to-vigorous physical activity, there is a noticeable improvement in muscle strength and body composition, positively affecting the positive emotions of older individuals and reducing trait anxiety [24]. Older adults engaging in two and a half hours of moderate-to-vigorous physical activity per week can reduce mortality by 22% [25]. It has significant effects on preventing and treating osteoporosis in postmenopausal women [26] and alleviating menopausal health anxiety [27]. Thirdly, in terms of mental health, moderate-to-vigorous physical activity not only reduces injury or improves function in elderly patients with hip fractures but also has positive effects on treating depression and phobias [28]. However, prolonged or excessively intense training may lead to increased levels of inflammatory mediators, potentially increasing the risk of injury and chronic inflammation [29] and even causing gastrointestinal distress [30].

Social anxiety refers to significant and persistent nervousness or fear in social situations or public settings, often leading to avoidance behaviors. The role of physical activity in alleviating symptoms of social anxiety has been widely studied and can be argued from two perspectives. From a dynamic perspective, the stress-buffering hypothesis suggests that positive factors can buffer the impact of external stress or other negative factors [31]. Engaging in physical activity allows individuals to experience pleasurable feelings and release negative emotions, reducing the level of social anxiety. From a theoretical perspective, researchers like Anderson and Shivakumar [3] have confirmed the positive impact of moderate-to-vigorous physical activity on reducing anxiety through physiological mechanisms. Ren and Li [31], using perceived social support as a mediating variable, confirm that perceived social support has a significant and positive impact on reducing social anxiety in Chinese rural left-behind children through physical activity. Currently, over 30 published narrative or meta-analysis reviews have examined the impact of physical activity on the structure of clinical or subclinical depression, social anxiety, self-esteem, emotional well-being, cognitive function, and sleep [32]. Among these, reducing social anxiety is achieved by increasing positive psychological resources within individuals, enhancing their adaptive abilities to external environments, and increasing opportunities for interpersonal interaction. Based on previous research, this study hypothesizes that moderate-to-vigorous physical activity will have a positive impact on reducing social anxiety.

**Hypothesis** **1** **(H1).** *Moderate-to-vigorous physical activity negatively influences social anxiety.*

Self-disclosure is the voluntary and intentional act of revealing one’s thoughts, feelings, or experiences to others [33]. It is an effective way of positively showcasing one’s thoughts and reinforcing self-confidence. High levels of self-disclosure have a certain impact on improving relationship quality, intimacy, and social adaptability [34]. Individual self-disclosure is influenced by the disclosure target, situation, and personal traits. Social penetration theory suggests that self-disclosure is a fundamental form of social exchange, and as people’s relationships develop, this social exchange becomes broader and more profound. Sports activities create an open environment for individuals to showcase themselves and let down their guard. Through physical contact in sports, individuals are prompted to engage in self-disclosure. Research conducted on experimental and control groups demonstrates a positive correlation between increased positive emotions and intimacy, with the willingness to engage in self-disclosure increasing with positive emotions during exercise. Cooper and Bowles [35] conclude from their experiments that physical contact activities can reduce the distance between individuals and increase the willingness to engage in self-disclosure. During sports activities, the body produces a significant amount of dopamine as physical intensity increases, leading to a pleasant mood, increased self-confidence, and more opportunities for self-disclosure. Based on previous studies, this research hypothesizes that moderate-to-vigorous physical activity has a positive impact on self-disclosure.

**Hypothesis** **2** **(H2).** *Moderate-to-vigorous physical activity positively affects self-disclosure.*

People often turn to social media to seek the attention and respect that they cannot obtain in real life, leading to two competitive hypotheses: social compensation and the rich get richer. Self-disclosure on social networks gradually replaces self-disclosure in interpersonal relationships because social media lacks visual cues. Therefore, individuals let go of their guard and be more proactive in self-disclosure to maintain online communication, contributing to the outbreak of online fraud. On social platforms, the open context allows individuals with high levels of social anxiety to vent their negative emotions and reshape their social image. However, this may also lead to an increase in anxiety levels as individuals cannot compensate for their anxiety through social media.

Social skills (companionship, self-control, self-disclosure, and adaptation) can affect people’s mental health, and they are negatively correlated with emotional expression and psychological distress [36]. On social media, individuals with low social anxiety have a stronger desire for self-disclosure and positive feedback [37]. Even when studying virtual individuals, it has been shown that people with social anxiety tend to disclose more private information [38]. Scholars, through research examining the relationship between the arousal of social anxiety in a tense interview environment and the expected self-disclosure, ultimately conclude that social anxiety and self-disclosure measures are negatively correlated [39]. In emotional relationships, social anxiety is one of the reasons for the lack of expression and the decline in the quality of romantic relationships [40]. Based on previous research, this study hypothesizes that self-disclosure has a positive impact on reducing social anxiety.

**Hypothesis** **3** **(H3).** *Self-disclosure negatively affects social anxiety.*

### 2.2. Self-Disclosure, Social Anxiety, and Social Avoidance

Social avoidance is a common psychological disorder among adolescents, representing a behavioral manifestation of social anxiety and social fear. It involves avoiding and refraining from normal social interactions, leading to interpersonal distancing, social loneliness, and anxiety [41]. From an animal perspective, social avoidance can be viewed as a survival technique or a submissive behavior, triggered by threats from dominant animals within the same species [42]. A literature review and analysis reveal the dual nature of social avoidance. On one hand, research by Fernández-Theoduloz et al. [43] found that social avoidance has a positive impact on curing depression. On the other hand, social avoidance is a major component of interpersonal issues, causing individuals to fear social interactions and to potentially use negative emotions as an outlet.

Based on previous research, negative emotions in various strata of society, as proposed by emotion regulation theory, may be mediated by avoiding emotional experiences and expressions. The disclosure of negative emotions is confirmed to be negatively correlated with specific events [44]. Individuals with psychological distress (loneliness, depression, low self-esteem, etc.) find it challenging to appropriately disclose personal information in new relationships and unstructured social situations [45]. Experimental interventions promoting self-disclosure have been found to reduce avoidance in breast cancer patients [34]. Most adults who stutter often use self-disclosure to reduce avoidance behaviors in social situations [46]. Therefore, this study hypothesizes that self-disclosure will have a positive impact on reducing social avoidance.

**Hypothesis** **4** **(H4).** *Self-disclosure negatively affects social avoidance.*

Social anxiety is considered an emotional state, while social avoidance is a direct behavioral avoidance of interpersonal interactions. According to Maslow’s hierarchy of needs, individuals desire acceptance and support from others while avoiding rejection. Social avoidance is crucial in social anxiety and social phobia, serving as a major factor in maintaining anxiety [47]. Experimental investigations indicate that social anxiety and social avoidance often emerge in early adolescence, and preventive or early intervention measures significantly reduce their occurrence [48]. The onset of social anxiety in adolescents may be partly attributed to an increase in avoiding social situations during this period [49] and familial genetic factors [50]. Kashdan et al. [51], through interacting with individuals with social anxiety, found a positive correlation between social anxiety and social avoidance, with a simultaneous display of highly sensitive behavior, avoiding closeness with others. Highly socially anxious participants in virtual reality CAVE systems tend to exhibit more sensitive avoidance behavior towards virtual people [52]. Social anxiety enhances avoidance behaviors related to facial features and expressions. Neutral faces are perceived as ambiguous social cues, strongly eliciting avoidance behaviors [53]. Based on previous research, this study hypothesizes that social anxiety will have a positive impact on enhancing social avoidance.

**Hypothesis** **5** **(H5).** *Social anxiety positively affects social avoidance.*

### 2.3. The Mediating Role of Social Anxiety and Self-Disclosure

In previous research, scholars often studied the level of self-disclosure and intimacy among adolescents on social media with social anxiety as a mediator. However, there has been minimal exploration of social anxiety and self-disclosure as mediating variables in examining relationships between other factors.

Shy children often adopt a sedentary lifestyle as a substitute for physical activity, displaying low social competence and social avoidance behavior, ultimately affecting the physical and mental health of children [54]. Li et al. [55] used mindfulness and social avoidance as mediating variables for physical activity and mental health, confirming a positive correlation between physical activity and mindfulness and mental health. It was negatively correlated with social avoidance and distress, indicating that successful sports events can effectively improve participants’ mental health by enhancing mindfulness levels, reducing social avoidance, and distress. Numerous studies demonstrate the positive effects of physical activity on treating psychological disorders (depression, anxiety, low self-esteem, etc.) and social challenges (avoidance, rumination, withdrawal, etc.). The more physical activity, the greater the therapeutic potential for psychological disorders [56]. Kagawa et al. [57] found that as the physical activity–pleasure index (PA-PI) increased, avoidance/rumination behaviors gradually decreased. Aerobic–anaerobic exercise can improve body composition and shape in obese women, enhance self-image and self-awareness, reduce social avoidance and distress, and achieve the goal of boosting self-esteem, confidence, and social skills [58]. Based on previous research, it can be inferred that there may be a negative correlation between physical activity and social avoidance. However, no scholars have explored the mediating effects. Therefore, this study hypothesizes that social anxiety and self-disclosure act as dual mediators in the relationship between moderate-to-vigorous physical activity and social avoidance.

**Hypothesis** **6** **(H6).** *Social anxiety and self-disclosure play a chain-like dual mediating role in the relationship between moderate-to-vigorous physical activity and social avoidance.*

**Figure 1:**
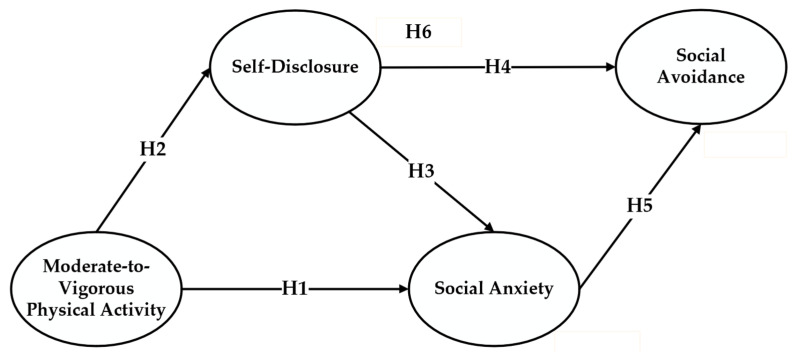
Hypothetical model.

Hypothesis 1 to Hypothesis 6 as shown in Figure 1.

## 3. Methods

### 3.1. Participants and Procedure

This study, conducted from July to August 2023, utilized both snowball sampling and convenience sampling methods to conduct a questionnaire survey among 500 adolescents from eight provincial key junior and senior high schools in the central China region, including 4 high schools and 4 junior high schools. The purposeful use of snowball sampling in collecting real data from adolescents enhances the specificity of the study and facilitates its application in analyzing special survey subjects, promoting scientific research. Convenience sampling, on the other hand, is convenient to operate and allows for the rapid acquisition of survey data, exhibiting hierarchy and representativeness in sample extraction, ultimately yielding a proportionate sample.

The selection of the central China region for this study is based on its inclusion of Hunan Province, Hubei Province, and Henan Province, which are among the top ten provinces in China for compulsory education. The random selection of provincial key junior and senior high schools expands the age and geographic representation of the study, making the results more representative.

Initially, researchers contacted school leaders and guardians, explaining the purpose and significance of the study, and distributed paper questionnaires to adolescents with their informed consent. Out of the 500 survey responses, 427 (including 189 from high school and 238 from junior high school) were considered valid, excluding incomplete, excessively brief, or duplicate data, resulting in an 85.4% response rate. Among these, 200 questionnaires were distributed to high school students, with 189 valid responses (94.5% response rate), and 300 questionnaires were distributed to junior high school students, with 238 valid responses (79.3% response rate).

According to the demographic data presented in Table 1, the sample consisted of 197 males (46.1%) and 230 females (53.9%). Among the participants, 40.0% were aged between 12 and 14, 36.1% were aged between 15 and 16, and 23.9% were aged between 17 and 18. Furthermore, 17.3% of respondents reported exercising for less than 1 h per day, 44.7% reported exercising for 1 to 2 h per day, and 37.9% reported exercising for more than 2 h per day. When asked about the past week, 30.9% of the surveyed individuals reported actively participating in moderate physical activities, 51.8% in strong physical activities, and 17.3% in very strong physical activities.

### 3.2. Measures

The questionnaire was designed with five sections. The first section involved the distribution of the questionnaire to collect basic information from the respondents, including their age, gender, and daily exercise duration. The purpose was to gain an understanding of the participants’ demographics.

Sections two through five were dedicated to collecting data on physical exercise, social anxiety, self-disclosure, and social avoidance, respectively. The measurement tools used were the exercise scale developed by Andersen et al. [59], the Liebowitz Social Anxiety Scale [60], DS Fusani’s Student Self-Disclosure Survey [61], and the Social Avoidance and Distress Scale (SAD) developed by Watson and Friend [41]. All four scales utilized a Likert 5-point scale for measurement, where response options ranged from 1 (strongly disagree, never) to 5 (strongly agree, always).

A pilot survey revealed deficiencies in language expression, distribution methods, and item arrangement within the scales. Consequently, the researchers made adjustments to some items. To ensure the reliability of the modified scales, a pre-test was conducted with 100 questionnaires distributed to students from eight provincially key middle and high schools in the central region of China, using convenience sampling. A total of 82 valid responses were received, and the Cronbach’s alpha coefficients were all above 0.8, confirming the rationality of the modifications made by the researchers.

### 3.3. Data Analysis

In this study, structural equation modeling (SEM) and AMOS 26.0 software were employed to analyze the constructed model. SEM is commonly used to assess latent variables in measurement models and test assumptions among latent variables in structural models [62]. The two-step modeling approach proposed by Anderson and Gerbing [63] was adopted, involving the evaluation of both measurement and structural models using SEM. Firstly, in the reliability and validity section, the researchers examined the reliability and validity of the measurement tools. The minimum Cronbach’s alpha coefficient for all variables was 0.841, indicating good reliability and validity of the instruments. Secondly, the researchers employed the maximum likelihood estimation method to validate the relationships among high-intensity physical activity, social anxiety, self-disclosure, and social avoidance. Thirdly, 5000 bootstrap samples were utilized to test the indirect effects between high-intensity physical activity and social avoidance. Lastly, the effectiveness of the model was assessed, and fit indices and path coefficients of the hypothesized model were measured.

## 4. Results

### 4.1. Assessment of the Measurement Model Reliability and Validity

Reliability and discriminant validity were assessed in this study by computing Cronbach’s alpha and composite reliability (CR) coefficients for latent variables [64]. The Cronbach’s alpha coefficients for all variables ranged from 0.841 to 0.871, and CR values were consistently above 0.80. The average variance extracted (AVE) for each variable fell within the range of 0.574 to 0.644 (see Table 2). Thus, all variables demonstrated high reliability and validity. Discriminant validity refers to the uniqueness of construct indicators. As shown in Table 3, the correlation coefficients between all variables were lower than the square root of AVE, indicating that the variables demonstrate satisfactory discriminant validity [65].

### 4.2. Common Method Variance

An examination was conducted to address the potential issue of common method variance (CMV) in the study. Firstly, the results of the Harman’s single-factor test indicated that the percentage of variance extracted from this unifactorial analysis was 40.495% (below the classical threshold of 50%), suggesting the absence of CMV [65]. Secondly, following the approach proposed by Mossholder et al. [66], a comparison between the confirmatory factor analysis (CFA) single-factor and multi-factor models was performed. The chi-square value for the single-factor model was 1432.3, with 90 degrees of freedom (df), while for the multi-factor model, it was 151.7, with 84 degrees of freedom (df). The ratio of the difference in chi-square values to the difference in degrees of freedom between the two models was 213.4. The significant difference in chi-square values between the two models indicated that the single-factor structure was not present. The impact of CMV on this study was deemed minimal and could be considered negligible.

### 4.3. Hypothesis Testing Results

Firstly, the error terms and residuals in the structural equation model did not exhibit negative values, indicating that the model did not violate assumptions. Secondly, the fit indices of the data to the structural equation model were high (χ^2^/df = 1.938, GFI = 0.976, AGFI = 0.928, NFI = 0.951, CFI = 0.976, TLI = 0.970), significantly surpassing the recommended values. Thirdly, the results of Pearson correlations, as shown in Table 4, indicated significant correlations between the independent variable, the mediating variable, and the dependent variable, supporting the validation of the hypotheses.

The World Health Organization has recently released the “2020 Guidelines on Physical Activity and Sedentary Behavior,” emphasizing that an average of 60 min of moderate-to-vigorous physical exercise per day is more beneficial for specific health outcomes in adolescents [67]. An analysis of the survey data revealed that the number of adolescents engaging in 60 min or more of physical exercise per day reached 353, accounting for a total percentage of 82.6% (see Table 1).

**Figure 2:**
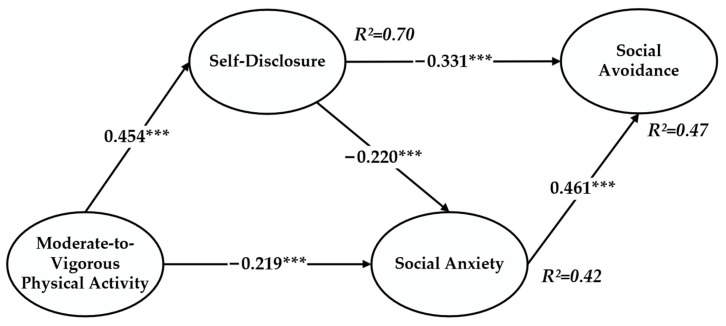
Structural path model. Note: *** *p* < 0.001.

The structural path model in Figure 2 revealed a significant positive correlation between moderate-to-vigorous physical activity and self-disclosure (*β* = 0.454, *p* < 0.001), supporting H1; a significant negative correlation between moderate-to-vigorous physical activity and social anxiety (*β* = −0.219, *p* < 0.001), supporting H2; a significant negative correlation between self-disclosure and social anxiety (*β* = −0.220, *p* < 0.001), supporting H3; a significant negative correlation between self-disclosure and social avoidance (*β* = −0.331, *p* < 0.001), supporting H4; and a significant positive correlation between social anxiety and social avoidance (*β* = 0.461, *p* < 0.001), supporting H5.

The researchers hypothesized that moderate-to-vigorous physical activity influences social avoidance through two mediators: self-disclosure and social anxiety. This study employed bootstrapping to test the mediating effects. The standardized results with a 95% confidence interval for 427 samples are presented in Table 4; the absolute value of the obtained Z score is 7.271, with no zero values within the 95% confidence interval. Furthermore, self-disclosure and social anxiety have a chain-mediated dual effect on the relationship between moderate-to-vigorous physical activity and social avoidance (standardized indirect effect = −0.349, *p* < 0.001), supporting H6. The findings indicate that adolescents engaging in more moderate-to-vigorous physical activity, exhibiting stronger self-disclosure, and experiencing lower social anxiety tend to have lower levels of social avoidance, and social anxiety and self-disclosure, as mediating variables, explain 47% of the variance in social avoidance.

## 5. Discussion

### 5.1. Theoretical Contribution

This study aims to explore the impact of moderate-to-vigorous physical activity on adolescent social avoidance from the perspective of objectification theory, contributing to theoretical analysis of social avoidance. Most existing research on social avoidance focuses on the reasons specific individuals exhibit social avoidance behavior, with limited studies on how to reduce such behavior [68]. This study specifically examines the relationship between moderate-to-vigorous physical activity and adolescent social avoidance, providing targeted insights and enriching relevant theoretical research.

In contrast to previous studies, this research first outlines the benefits of adolescents engaging in moderate-to-vigorous physical activity and the reasons behind adolescent social avoidance. It then establishes and discusses the relationships among moderate-to-vigorous physical activity, social anxiety, self-disclosure, and social avoidance. Finally, it argues for the mediating effects of social anxiety and self-disclosure on the relationship between moderate-to-vigorous physical activity and social avoidance. The results indicate mutual influences among moderate-to-vigorous physical activity, social anxiety, self-disclosure, and social avoidance.

Furthermore, the findings show that as the metabolic equivalent (MET) reaches 3, the level of anxiety in adolescents gradually decreases. This is consistent with the views of Sallis et al. [20], indicating that the longer adolescents engage in moderate-to-vigorous physical activity, the lower the probability of experiencing symptoms of social anxiety. When the metabolic equivalent (MET) reaches 3.5, adolescents’ psychological defenses gradually weaken, demonstrating a strong desire for communication and expression. Their self-disclosure behaviors become more pronounced.

Firstly, this study further confirms the negative impact of moderate-to-vigorous physical activity on social anxiety, consistent with the findings of Ren and Li [31] (supporting Hypothesis 1). It innovatively suggests that moderate-to-vigorous physical activity positively influences self-disclosure, supported by empirical evidence from psychological and sports training perspectives (supporting Hypothesis 2). Through the literature support and research data, a negative correlation between self-disclosure and social anxiety is revealed, in line with the research of Wang and Sugiyama [36] (supporting Hypothesis 3). Overall, the study suggests that moderate-to-vigorous physical activity enhances the willingness for self-disclosure and reduces the level of social anxiety, emphasizing the importance of adolescents participating in moderate physical activities for holistic well-being.

Secondly, the study discovers certain relationships among self-disclosure, social anxiety, and social avoidance. Self-disclosure negatively affects social avoidance, a finding that has been confirmed in research, whether in maintaining romantic relationships or expressing oneself among family members [40], while social anxiety positively affects social avoidance. Compared to directly demonstrating the relationship between independent and dependent variables, this study indirectly illustrates the impact of social anxiety and self-disclosure on social avoidance, further exploring the influence of moderate-to-vigorous physical activity on social avoidance.

Finally, the study proposes a hypothetical path with social anxiety and self-disclosure as mediators, delving into the relationship between moderate-to-vigorous physical activity and social avoidance. It offers an in-depth exploration of the reasons individuals exhibit avoidance behaviors from the perspective of psychological changes induced by physical activity.

### 5.2. Practical Implications

Adolescents are in a crucial period of growth and development. The emergence of social issues is detrimental to their psychological well-being, leading to heightened negative self-perception and negative information processing biases. Based on the research findings, it is evident that moderate-to-vigorous physical activity has a positive impact on reducing social anxiety levels and enhancing the willingness for self-disclosure among adolescents. Moreover, social anxiety and self-disclosure act as intermediaries in the relationship between moderate-to-vigorous physical activity and social avoidance behaviors in adolescents.

To address these issues comprehensively, a collaborative effort from the government, society, schools, and families is essential. It is crucial to provide opportunities for adolescents to engage in physical activities and ensure the availability of basic sports facilities and conditions.

Specific recommendations are as follows:

First, increase opportunities and motivation for adolescent physical exercise. The positive effects of adolescent participation in physical exercise on both physiological and psychological health development are well-established. The government should actively introduce relevant policies to promote adolescent participation in physical exercise, allocating resources for sports facilities and equipment. For instance, an annual allocation of funds for sports equipment could ensure that every adolescent has the opportunity to engage in physical exercise. Communities should provide safe, diverse, and comprehensive social practice platforms, such as organizing community sports events and family fun competitions and effectively utilizing public sports facilities. Schools should actively offer extracurricular physical education courses, encouraging and organizing adolescents to participate in various sports competitions, such as class sports festivals, grade-level sports meets, and club challenges.

Second, encourage adolescents to actively overcome negative emotions and dare to express themselves. Positive emotions contribute to enhancing immunity and self-confidence, and strengthening communication with the outside world can reduce the occurrence of negative emotions such as depression, anxiety, and avoidance, promoting overall physical and mental health. Therefore, the government should increase its focus on the mental health of adolescents, garnering support for mental health education in society and higher education institutions. The establishment of an integrated adolescent sports network involving schools, communities, and families is essential. Schools should organize lectures on adolescent mental health education, guide students to face social issues, enhance their social skills, and establish dedicated psychological counseling rooms. Families should create a warm and harmonious atmosphere, strengthen communication with children and schools, promptly understand children’s psychological performance at school, and pay attention to their psychological health development, achieving a collaborative education effect between home and school. Adolescents should cultivate correct social concepts, learn to recognize, accept, and relax themselves, identify negative emotions and cognitions, master emotional regulation skills, indirectly build self-confidence by exploring their interests or strengths, actively seek professional help, and engage in regular moderate-to-vigorous physical exercise.

Third, develop a “Physical Activity + Mental Health” monitoring system. In the digital age, administrators of such systems can utilize data on the frequency, duration, and other aspects of adolescents’ participation in physical exercise [69]. This can be used to compare the impact on adolescents’ social skills before and after engaging in physical exercise, further validating the accuracy of the study’s results and exploring the influence of physical exercise on other psychological issues in adolescents.

In conclusion, it is imperative for the government, society, schools, and families to align with the national goal of building a strong sporting nation and strengthen the physical health of adolescents. This study not only reveals the health and social issues faced by contemporary Chinese adolescents but also provides a theoretical basis for creating a conducive social environment and enhancing physical and mental health for adolescents.

### 5.3. Limitations

This study has certain limitations. Firstly, there is the singularity of the mediating variables; this study selected only social anxiety and self-disclosure as the mediating variables to explore the mechanism through which moderate-to-vigorous physical activity regulates adolescent social avoidance. Future research could gradually analyze multiple variables to identify the optimal mediating variables and provide scientifically grounded recommendations for addressing the health and growth issues of adolescents.

Secondly, there is the limitation of the study’s subjects. Variations in adolescents’ age, gender, and geographic location can influence the degree of social avoidance behavior. Future research could further refine and deepen the analysis based on age, gender, and geographic location, expanding the sample size and range to enhance the external validity of the study. This could be achieved, for example, by conducting research on adolescents aged 12 to 18 of different genders from all 34 provincial-level administrative regions in China or including international adolescents to test the accuracy and applicability of the study results.

Thirdly, there is a limitation regarding the accuracy of the research methodology. This study is a cross-sectional study. To more accurately and effectively reflect the impact of moderate-to-vigorous physical activity on adolescent social avoidance, future research might consider setting up experimental and control groups and adopting a longitudinal study design.

## 6. Conclusions

Current cross-sectional survey research indicates that moderate-to-vigorous physical activity has a positive impact on the social avoidance behaviors of adolescents. Specifically, the influence of moderate-to-vigorous physical activity on social avoidance is mediated by two variables: social anxiety and self-disclosure. Regular physical activity among adolescents reduces social anxiety levels, increases the willingness for self-disclosure, and consequently reduces social avoidance behaviors.

Based on these findings, the study suggests a coordinated approach involving the government, society, schools, and families to enhance the physical and mental well-being of adolescents. This collective endeavor is geared toward establishing a strong and diverse talent reservoir for national progress. By fostering and facilitating the active engagement of adolescents in physical activities, this initiative not only enhances the overall health of the younger generation but also bolsters the nation’s advancement by cultivating a varied and resilient talent pool.

## Figures and Tables

**Table 1:** Participant profile (*n* = 427).

Profiles	Survey (%)
**Age**	
12–14	171 (40.0%)
15–16	154 (36.1%)
17–18	102 (23.9%)
**Gender**	
Male	197 (46.1%)
Female	230 (53.9%)
**Exercise for a long time every day**	
Less than 1 h	74 (17.3%)
1–2 h	191 (44.7%)
More than 2 h	162 (37.9%)
**Participation in physical exercise in the past week**	
Moderate physical activities	132 (30.9%)
Strong physical activities	221 (51.8%)
Very strong physical activities	74 (17.3%)

**Table 2:** Reliability and validity testing.

Items	Loadings	Cα	AVE	CR
Moderate-to-vigorous physical activity (MVPA)		0.841	0.644	0.844
MVPA1	0.837			
MVPA2	0.832			
MVPA3	0.735			
Self-disclosure (SD)		0.871	0.634	0.873
SD1	0.856			
SD2	0.841			
SD3	0.691			
SD4	0.787			
Social anxiety (SAN)		0.868	0.628	0.870
SAN1	0.723			
SAN2	0.810			
SAN3	0.775			
SAN4	0.855			
Social avoidance (SAV)		0.842	0.574	0.843
SAV1	0.772			
SAV2	0.782			
SAV3	0.706			
SAV4	0.768			

Note: Cα = Cronbach’s alpha; AVE = average variance extracted; CR = composite reliability; MVPA = moderate-to-vigorous physical activity; SD = self-disclosure; SAN = social anxiety; SAV = social avoidance.

**Table 3:** Discriminant validity test.

Construct	MVPA	SD	SAN	SAV
MVPA	**0.802**			
SD	0.413 **	**0.762**		
SA	−0.366 **	−0.371 **	**0.910**	
SAV	−0.419 **	−0.454 **	0.454 **	**0.782**

Note: the square root of the average variance extracted (AVE) is in the diagonal (bold); off-diagonal elements represent Pearson correlations. MVPA = moderate-to-vigorous physical activity; SD = self-disclosure; SAN = social anxiety; SAV = social avoidance. ** *p* < 0.01.

**Table 4:** Indirect effects.

	Point Estimate	Product of Coefficients		Bootstrapping		
				Bias-Corrected 95% CI		Two-Tailed Significance
		*SE*	*Z*	Lower	Upper	
**Indirect Effects**						
MVPA→SAV	−0.349	0.048	−7.271	−0.443	−0.254	**

Note: standardized estimates based on 5000 bootstrap samples. SE = standard error; MVPA = moderate-to-vigorous physical activity; SAV = social avoidance. ** *p* < 0.01.

## Data Availability

The data presented in this study are available on request from the corresponding author. The data are not publicly available due to compliance with informed consent and ethical considerations.
